# The crucial role of explainable artificial intelligence (XAI) in improving health care management

**DOI:** 10.1007/s10729-025-09720-y

**Published:** 2025-09-30

**Authors:** Arne Johannssen, Nataliya Chukhrova

**Affiliations:** 1 Harz University of Applied Sciences, Faculty of Business Studies, 38855 Wernigerode, Germany; 2 University of Hamburg, Hamburg Center for Health Economics, 20354 Hamburg, Germany; 3 University of Heidelberg, Faculty of Medicine Mannheim, Department of Health Economics, 68167 Mannheim, Germany

**Keywords:** Artificial intelligence (AI), Ethical AI, Explainability, Interpretability, Medical decision-making, Quality improvement, Responsible AI, Transparency, Trustworthy AI, XAI

## Abstract

This current opinion explores the transformative potential of explainable artificial intelligence (XAI) for health care management systems. While AI has already demonstrated substantial benefits in clinical decision-making, operational efficiency and patient outcomes, its adoption is often hindered by the lack of transparency in AI-driven decision-making. XAI bridges this gap by providing interpretability, thereby increasing trust between policy-makers, clinicians, administrators and patients. However, despite promising examples, the explicit integration of XAI remains underexplored in health care management research. This current opinion therefore aims to emphasize the crucial role of XAI in improving health care management and to position it as an important topic for advancing the field, with Health Care Management Science (HCMS) playing a leadership role in fostering this development.

## Introduction

Explainable artificial intelligence (XAI) has substantial transformative potential to improve the quality of health care systems worldwide. AI has proven to be a powerful tool in all areas of health care, including clinical decision-making, operational management, patient outcomes and health monitoring [20, 34]. XAI, as a framework designed to ensure interpretability and accountability in AI-driven decisions, plays a central role in ensuring that the adoption of AI directly supports quality improvement efforts [13]. Quality in health care is multidimensional, covering the key pillars safety, effectiveness, patient-centeredness, timeliness, efficiency and equity. As AI is increasingly integrated into health care processes, from predictive modeling to workflow optimization, it is critical that the technology aligns with these globally recognized dimensions of quality defined by the Institute of Medicine (IOM) [17].

XAI fills a critical gap by making “black box” AI systems interpretable to a wide range of stakeholders (like clinicians, administrators, patients) and ensuring that decisions are not only accurate, but also fair, reliable, unbiased and reasonable [3]. While fairness, reliability and ethical alignment are widely accepted goals in AI deployment, these cannot be assured solely through performance metrics or regulatory compliance. Explainability serves a unique role by enabling stakeholders to question how and why a model arrives at certain decisions [9]. This transparency helps detect hidden biases, fosters appropriate trust calibration (i.e., neither over- nor under-reliance on AI) and supports accountability [35]. In this sense, XAI complements other essential safeguards like human oversight, validation procedures and governance frameworks.

This current opinion ties in with a fundamental discussion about the appropriate role of explainability versus interpretability. Rudin [28] argued that in high-stakes decision-making, such as health care, one should forego complex, opaque models altogether and instead prioritize inherently interpretable models from the outset. This view challenges the premise that post-hoc explanation of black-box models is sufficient or even desirable. However, we are confident that post-hoc explainability techniques will play a central role in supporting transparency and accountability, particularly where interpretable models may underperform or be infeasible due to data complexity.

Thus, the aim of this current opinion is to emphasize the crucial role of XAI in improving health care management and to highlight its potential as a priority topic for advancing research in the field, with *Health Care Management Science* (HCMS) positioned to play a leading role. To this end, the taxonomy of XAI (Section 2) and relevant publications in HCMS (Section 3) are briefly described first. Then the multiple potential of XAI to support the key pillars of quality in health care is discussed and exemplified by illustrative scenarios (Section 4). The article concludes with recommendations to advance the discourse on XAI in HCMS (Section 5).

## Taxonomy of XAI

There are several taxonomies of approaches to XAI, be it on the basis of interpretability vs. explainability, global vs. local explanations or model-agnostic vs. model-specific explainability. The most fundamental breakdown of XAI is into *interpretable models* and *explainable models* [36]. Not all statistical methods are inherently interpretable, but many – such as linear/logistic regression, decision trees, Bayesian models – are generally considered easy to understand and explain. These are examples of interpretable models, meaning their internal logic is transparent by design. In contrast, more complex models like neural networks or ensemble methods (e.g., random forests, gradient boosting) are often considered “black boxes” and require post-hoc explainability techniques [22, 28]. The term *post-hoc explainability* covers all methods that provide explanations after the training of a machine learning (ML) model, which can be divided into two categories: model-agnostic and model-specific methods [2, 5]. *Model-agnostic methods* are explanation techniques that can be applied to any ML model, while *model-specific methods* are tailored to the architecture or structure of a specific ML model. The benefits of implementing model-specific methods include a deeper understanding of the mechanisms underlying the decision-making process by examining the inner workings of the model and developing a more customized, explainable model [2, 5, 21]. Table 1 gives an overview of common XAI techniques.

**Table 1:** Overview of common XAI techniques [5, 29]

Category	Method	Description	Example models / use cases
Interpretable models	Linear/Logistic regression	Models whose parameters have direct, transparent interpretations	Risk scoring, resource planning
	Decision trees	Tree-based logic flows for classification or regression	Triage rules, patient segmentation
	Bayesian models	Probabilistic models with transparent priors and inference steps	Uncertainty estimation, diagnostics
Model-agnostic methods	Local Interpretable Model-agnostic Explanations (LIME)	Approximates black-box predictions locally with simple interpretable models	Any black-box classifier or regressor
	SHapley Additive exPlanations (SHAP)	Uses game theory to assign feature importance based on marginal contribution	Tree-based models, neural networks
	Counterfactual explanations	Shows how small changes to inputs could alter model decisions	Clinical eligibility, policy decisions
Model-specific methods	Feature importance (e.g., permutation)	Measures decrease in performance when features are altered or removed	Random forests, XGBoost
	Activation analysis	Examines neuron activation patterns to interpret outputs	Deep neural networks (CNNs, RNNs)
	Attention weights	Highlights input components most attended to by the model	Transformer models, NLP tasks

## Emerging work on XAI in HCMS: gaps and opportunities

The important role of XAI has not yet been sufficiently emphasized in health care management research, including HCMS. Even though there are several publications on model-agnostic and model-specific methods in HCMS, especially since 2023, hardly any article explicitly thematizes XAI, not even current review papers [25, 33]. Table 2 summarizes recent key studies published in HCMS that incorporate XAI techniques or touch on interpretability in complex AI applications. Although several papers apply post-hoc methods such as SHAP or feature importance, most do not explicitly frame their contributions in terms of XAI. This suggests a gap in conceptual engagement with explainability, even when relevant methods are used. Moreover, no studies in HCMS appear to use LIME, counterfactual explanations (in the XAI sense), activation analysis or attention weights to date.

While the studies mentioned in Table 2 show emerging interest in explainability methods, there is little exploration of the broader implications of XAI in health care management decision-making. The lack of foundational or critical perspectives on XAI may reflect a broader uncertainty about how to reconcile the interpretability-performance trade-off, regulatory constraints and the diverse needs of decision-makers in this space. This raises important questions:

- What kinds of decisions in health care management require explainability?
- In what contexts might inherently interpretable models be sufficient or preferable?
- How should health care managers weigh predictive performance against clarity and transparency?

**Table 2:** Summary of recent XAI-related studies in HCMS

Study	XAI method	Use case
Bartenschlager et al. [6]	General discussion	COVID-19 triage algorithms in emergency department (ED)
Zilker et al. [36]	Model transparency	ICU admission prediction (sepsis symptoms)
Ahmed et al. [1]	SHAP	Factors behind left against medical advice (LAMA)
Bertsimas et al. [7, 8]	SHAP	COVID-19 risk and treatment optimization
Seo et al. [31]	SHAP	Discharge prediction in ED
Shin et al. [32]	SHAP	Outpatient wait time prediction
Caserta & Romero [10]	Feature importance	Predicting surgery durations
Demiray et al. [12]	Feature importance	Classifying patients by activation level
Dunstan et al. [14]	Feature importance	Predicting no-show appointments
Elitzur et al. [15]	Feature importance	Optimal test admission under resource constraints
Pall et al. [26]	Feature importance	Factors associated with drug shortages
Rao et al. [27]	Feature importance	Antibiotic adherence prediction
Schäfer et al. [30]	Feature importance	Operational patient-bed assignment problem

## XAI and the quality of health care

In the following, we examine how XAI can contribute to improving each of the six core pillars of health care quality defined by the IOM: safety, effectiveness, patient-centeredness, timeliness, efficiency and equity [17]. For each dimension, we define its scope and provide a real-world-inspired scenario that illustrates the potential of XAI to support and strengthen quality improvement.

*Safety* refers to avoiding harm to patients from the care that is intended to help them. In practice, this includes minimizing diagnostic and treatment errors, adverse events and risks introduced by clinical technologies and/or workflows. As AI models are increasingly used in clinical decision-making, their opacity can obscure sources of error, which makes explainability essential for safe implementation [4, 13, 16].

### Illustrative scenario 4.1

(Safety) 

When predicting adverse events like post-surgical complications, a hospital employs a complex ensemble model. SHAP values are used to explain individual risk predictions by showing which features (like abnormal lab values or comorbidities) contributed most to a given output. This allows clinicians to validate predictions against clinical context and to recognize when the model may be misled by outliers or missing data, which will reduce harm caused by overreliance on flawed outputs.

*Effectiveness* involves delivering care that is based on sound scientific evidence and that maximizes the likelihood of desired health outcomes. It requires the avoidance of both overuse and underuse of medical services, and tailoring care based on clinical need rather than availability or habit. XAI can help clinicians and administrators understand whether AI-driven recommendations align with evidence-based guidelines [3, 13, 23].

### Illustrative scenario 4.2

(Effectiveness) 

A hospital system deploys an AI model to recommend imaging for suspected stroke cases. By applying SHAP globally and LIME locally, the model reveals that some recommendations are overly sensitive to irrelevant patient history variables, such as remote history of childhood asthma or past minor orthopedic injuries. In practice, the clinical team identifies these variables as irrelevant based on face validity and domain expertise, confirming that these factors should not influence imaging decisions for stroke suspicion. Using this insight, the team adjusts thresholds and input features, which leads to more targeted magnetic resonance imaging (MRI) usage. This improves adherence to evidence-based protocols while reducing unnecessary imaging, and leads to enhanced clinical effectiveness.

*Patient-centeredness* requires that care decisions take into account patients’ individual preferences, needs and values [12]. AI systems must be able to be interpreted not only by clinicians, but also by patients themselves to ensure that patients are informed participants in their care. XAI can improve shared decision-making by explaining AI recommendations in a way that patients can understand [11]. Since the recommendations are often binary or multiple outcomes where only one decision is made, XAI supports the understanding of how the corresponding probabilities were generated.

### Illustrative scenario 4.3

(Patient-centeredness) 

An oncology clinic uses an AI system to estimate the success probabilities of different chemotherapy regimens. To enhance shared decision-making, counterfactual explanations are employed to illustrate how modifiable factors, such as medication adherence or maintaining consistent physical activity during treatment, could influence predicted outcomes. These factors can be realistically adjusted by patients during treatment, allowing them to see how their actions may impact treatment effectiveness. This empowers patients to participate more actively in their care decisions and fosters trust in AI-driven recommendations.

*Timeliness* ensures that patients receive care without unnecessary delays. Delays in diagnosis, treatment or administrative decisions can increase patient stress and reduce system efficiency [31, 32]. XAI can play a crucial role in identifying the drivers of delays and informing real-time prioritization decisions.

### Illustrative scenario 4.4

(Timeliness) 

In a busy ED, a wait-time prediction model is integrated into patient triage. XAI tools such as SHAP explain which factors (vital signs, arrival time, chief complaint, among others) most influence predicted delays. This transparency helps staff optimize resource allocation and adjust triage flow dynamically. It also enables better communication with patients about expected waiting times and thus leads to reduced dissatisfaction.

*Efficiency* refers to avoiding waste, including waste of equipment, supplies, time, effort, ideas, energy and financial resources. Efficient systems deliver high-quality care using the least amount of unnecessary input without compromising outcomes [15, 30]. XAI can support this by revealing how models make resource-related decisions, which in turn helps organizations understand and refine cost-saving measures.

### Illustrative scenario 4.5

(Efficiency) 

A hospital uses a random forest based system to optimize ICU staffing. Initially, staff distrusts the system’s recommendations. By applying feature importance as model-specific explanation technique, it becomes clear that the system accounts for historical trends in patient acuity and seasonal variation. With this insight, administrators can adopt the system’s suggestions, reduce overstaffing and save costs without compromising care quality. Moreover, providing clear explanations of how the system generated staffing recommendations was essential in convincing senior managers of the tool's value and reliability prior to purchase and deployment.

*Equity* requires that all patients receive fair and consistent care regardless of their gender, ethnicity, geographic location or socioeconomic status. AI models can inadvertently pass on biases in historical data, thus reinforcing existing inequalities. XAI provides the tools needed to detect, assess and eliminate such biases, assuring that health care decisions are fair and equitable [3, 18].

### Illustrative scenario 4.6

(Equity) 

A hospital deploys an AI system to prioritize patients on a surgery waiting list. SHAP values reveal that urban ZIP codes are contributing disproportionately to higher prioritization scores, due to historical data bias. Combined with group fairness auditing, this insight leads to a retraining process that eliminates geographic bias and improves fairness in access to surgical services.

The above scenarios illustrate the potential of XAI to enhance each pillar of health care quality. However, it should be noted that explanation techniques such as SHAP, LIME or counterfactuals do not always guarantee faithful or causally valid interpretations and may sometimes oversimplify complex clinical or operational realities [3]. In certain contexts, they may introduce new uncertainty, for example, by highlighting spurious correlations or suggesting individual responsibility in ethically sensitive cases [5, 9]. Moreover, the usefulness of explanations depends not only on technical validity but also on how well they are understood and trusted by diverse stakeholders [11, 13]. As such, health care professionals and decision-makers should remain aware while explainability is valuable, it is not a universal solution and needs to be applied thoughtfully and critically in the broader context of health care.

## Advancing XAI in health care management: the role of HCMS

The advantages of XAI are evident, but its implementation entails many challenges. Finding the right balance between explainability and predictive accuracy remains an ongoing concern [18]. Ensuring that explanations make sense to different stakeholders requires careful design and customization. Furthermore, integrating XAI into existing health care workflows and regulatory frameworks requires robust standardization and validation efforts. Despite these challenges, the potential of XAI to revolutionize health care management is immense. By making AI systems more transparent, equitable and actionable, XAI is directly aligned with the core mission of HCMS: to publish research dealing with health care delivery, health care management, and health care policy. Therefore, we conclude with suggestions to realize the potential of XAI in health care management research, with HCMS well positioned to lead these efforts within the field:

- Authors could examine the trade-offs between implementing inherently interpretable models versus post-hoc explainability techniques. Highlighting case-specific benefits would provide guidance to practitioners on which approach to take in different circumstances and also advance the fundamental debate initiated by Rudin [28].
- Future studies in HCMS can explore which types of decisions in health care management (staffing, scheduling, discharge planning, cost estimation...) most critically require explainability, and under what circumstances stakeholders demand transparent reasoning.
- Authors should be encouraged to follow established reporting guidelines that promote transparency and explainability in AI applications. For instance, the EQUATOR Network lists several relevant frameworks like TRIPOD-AI, CONSORT-AI, SPIRIT-AI, which include items related to interpretability and explainability [19, 24].
- HCMS may publish papers examining how XAI aligns with existing health care regulations, ethical standards and frameworks like the General Data Protection Regulation (GDPR) and the Health Insurance Portability and Accountability Act (HIPAA).
- The journal could encourage submissions that contribute to developing domain-specific guidelines (e.g., clinical and operational domains) for applying XAI in health care. This includes standardizing interpretability metrics, validation protocols as well as workflows for integrating XAI into clinical practice.
- Future publications may also propose ways in which health care professionals can learn to understand and use XAI effectively. Bridging this gap is essential for the successful adoption of XAI in practice.

## Data Availability

No new data were created or analysed, so data sharing is not applicable to this article.
